# Effects of Affect and Source on Adoption of Health Information

**DOI:** 10.1002/pchj.70109

**Published:** 2026-05-28

**Authors:** Rui Shi, Ziyu Chen, Junchen Shang

**Affiliations:** ^1^ School of Economics and Management Yanshan University Qinhuangdao China; ^2^ Department of Medical Humanities, School of Humanities Southeast University Nanjing China

**Keywords:** adoption intention, affect, health information, information source

## Abstract

We explored how affective states, information affect, and source impacted health information adoption. Adoption of expert‐sourced information was greater than that from GAI, in line with the source credibility framework. In negative or neutral states, or when receiving content from GAI, people preferred positive over negative information, consistent with the affect‐as‐information hypothesis.

Nowadays, attention to health information is a common phenomenon. Some information emphasizes benefits (positive), while other clarifies risks (negative) (Carpenter et al. 2010). Affect influences information adoption intention, though controversies exist. As the affect‐as‐information hypothesis (Clore and Huntsinger 2007), positive affect (PA) strengthens the readily accessible cognitive tendencies and promotes heuristic processing, thereby increasing the perceived credibility and adoption. However, negative affect (NA) promotes analytic strategies, which may reduce information adoption. Nonetheless, the resource allocation model (Ellis et al. 1997) suggests both PA and NA facilitate heuristic strategies, which may increase information adoption. These theories do not classify the effects of information affect and people's affective state. Although some research partially supports affect‐as‐information that people tend to trust positive rather than negative information (Berger and Milkman 2012), it is unclear whether affective state can modulate the effect of information affect.

Recently, advanced technologies such as Generative AI (GAI) improve the spread of health information, while the undisclosed training details have raised concerns about algorithm bias (Van Dis et al. 2023), which cause negative attitudes toward GAI. According to the source credibility framework, professional expertise shapes public trust in health messages (Vafeiadis 2023). However, whether people tend to adopt health information from human experts than GAI is unclear.

In sum, both affect and information source may impact adoption, whether their interaction remains unknown. This study asked participants to evaluate health information (positive or negative) from different sources (human medical experts or GAI) under different states. We hypothesize that source may modulate the effect of affect on health information, such that people may trust information from human experts, as a halo effect regardless of affect, while people may rely on affect to judge information from GAI. Another exploratory hypothesis is an interaction between affective state and information valence, with no specific predictions about the trends.

This experiment was approved by the Ethics Committee of Qinhuangdao First Hospital. One hundred and sixty‐five younger adults (aged 18–30, 82 males) were recruited through ‌Credamo and randomly assigned to positive/negative/neutral group. This study employed a 3 (affective state: positive/negative/neutral) × 2 (source: GAI/medical expert) × 2 (information affect: positive/negative) mixed design, with affective state as between‐participant variable. Six information sources (3 GAI/3 medical experts) and 12 pairs of information (12 positive/12 negative) were selected (see Supporting Information for manipulation checks for familiarity, valence ratings, credibility, and perceived risk).

Participants first completed the Positive and Negative Affect Schedule (PANAS) (Watson et al. 1988). For each question, participants were asked to rate the current affective state on a 5‐point scale (1 = *very slightly or not at all*, 5 = *very much*) (Watson et al. 1988). Then participants watched a video for no less than 128 s (see Supporting Information for detailed information) and completed PANAS again. The formal experiment followed the affect induction. In each trial, one information source was presented above one piece of information. Participants were required to rate adoption intention using a 5‐point Likert scale (1 = *strongly disagree*, 5 = *strongly agree*) with no time limit. Twenty‐four non‐repetitive “source‐information” sets were presented in a random sequence.

Paired‐samples *t*‐tests were conducted on the scores of PA and NA before and after affect induction. The positive group reported higher overall PA (*t*(54) = −5.516, *p* < 0.001, *d* = 0.527) and lower overall NA (*t*(54) = 6.343, *p* < 0.001, *d* = −0.668) after induction. The negative group reported lower overall PA (*t*(54) = 13.523, *p* < 0.001, *d* = −1.699) and higher overall NA (*t*(54) = −5.896, *p* < 0.001, *d* = 0.704) after induction. The neutral group showed no significant changes for either PA (*t*(54) = 1.700, *p* = 0.095, *d* = −0.191) or NA (*t*(54) = 1.731, *p* = 0.089, *d* = −0.249) after induction.

A repeated‐measures ANOVA was performed on ratings of adoption. The main effects of information affect and source were significant, *F*(1,162) = 10.557, *p* = 0.001, *η*_*p*_^2^ = 0.061, *F*(1,162) = 76.551, *p* < 0.001, *η*_*p*_^2^ = 0.321. The interaction between information affect and source was significant (Figure 1A), *F*(1,162) = 4.456, *p* = 0.036, *η*_*p*_^2^ = 0.027. Simple effect analysis showed regardless of information affect, people preferred information from medical experts rather than GAI, *F*s ≥ 53.682, *p*s < 0.001. People preferred positive information when source was GAI, *F*(1,162) = 15.025, *p* < 0.001, *η*_*p*_^2^ = 0.084. The interaction between information affect and affective state was significant (Figure 1B), *F*(2,162) = 3.647, *p* = 0.028, *η*_*p*_^2^ = 0.043. Simple effect analysis showed people preferred positive information in negative and neutral states, *F*s ≥ 4.292, *p*s < 0.050, *η*_*p*_^2^ ≥ 0.026. Other effects were not significant, *F*s > 1.102, *p*s > 0.1.

**FIGURE 1:**
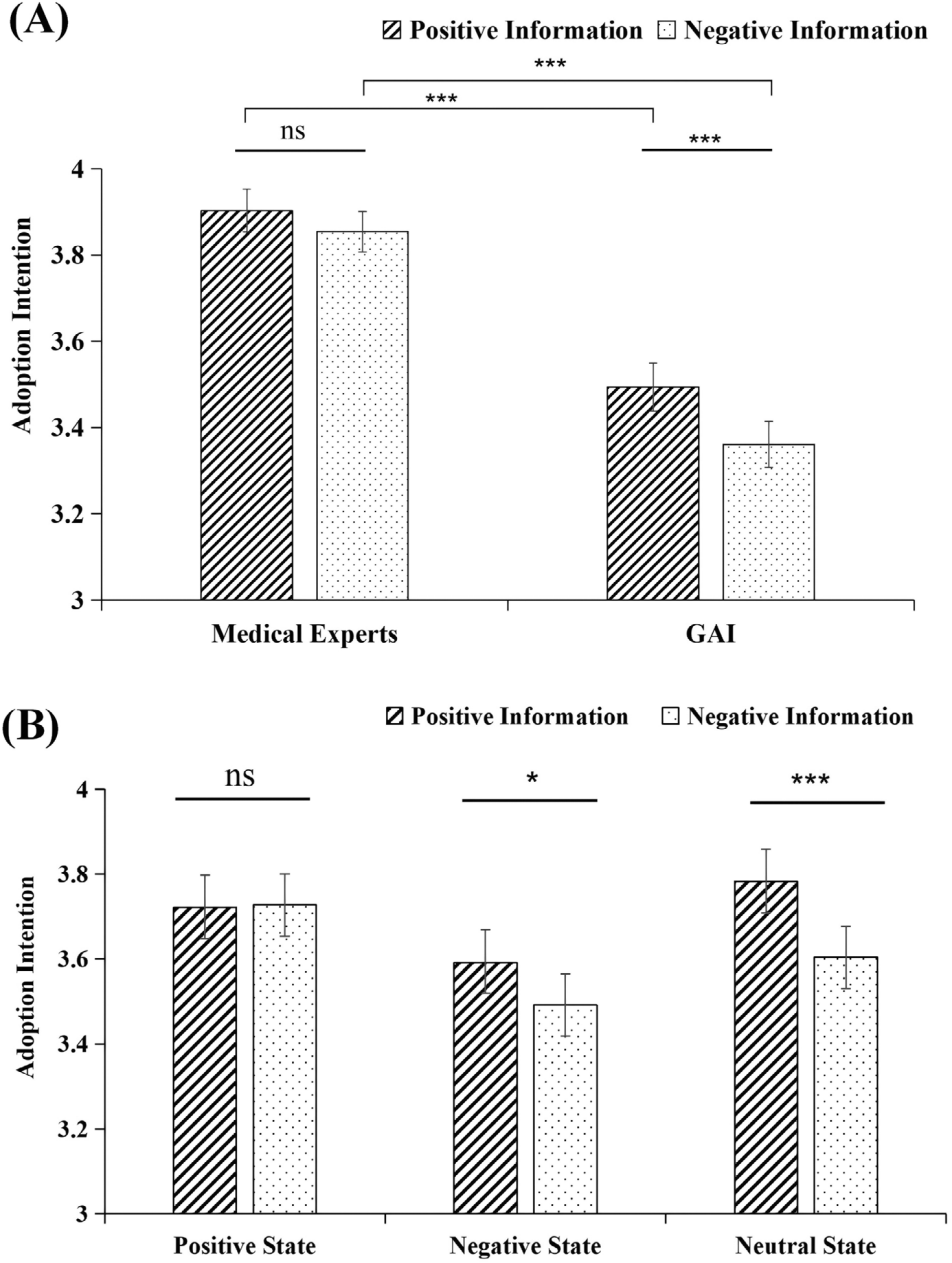
(A) Mean adoption intention as a function of information sources and information affect. (B) Mean adoption intention as a function of affective state and information affect. The error bars represent standard errors. *p < 0.05, ***p < 0.001, ns, non‐significant.

People preferred health information from medical experts rather than GAI, suggesting that the professional expertise of human experts resulted in a halo effect, consistent with the source credibility framework (Vafeiadis 2023). Moreover, GAI's algorithmic bias and opacity (Van Dis et al. 2023) trigger people's distrust and defensive information processing, reducing the information adoption intention. Additionally, people took information affect as a cue to judge messages from GAI since positive messages may ease distrust toward GAI and raise acceptance, whereas negative messages amplify skepticism.

Our findings align with the affect‐as‐information hypothesis (Clore and Huntsinger 2007) rather than the resource allocation model (Ellis et al. 1997). We showed a priority of positive state on adoption intention. Participants in positive state showed no bias toward positive and negative information, supporting the affect‐as‐information hypothesis (Clore and Huntsinger 2007), such that positive state increases heuristic strategy and gullibility. However, in both negative and neutral states, participants preferred positive information, aligning with Berger and Milkman (2012)'s. On the one hand, the neutral state represented a baseline where other cues became more influential, such that positive information elicited heuristic processing whereas negative information elicited analytic processing, as the affect‐as‐information hypothesis suggested (Clore and Huntsinger 2007). On the other hand, although the negative state caused analytic processing, it did not overcome the effect of positive information, consistent with Bazarova et al. (2013)'s assertion that people prefer to share positive affect than NA on Facebook.

There are two limitations. We used highly simplified, fixed‐length short sentences, which differ from the complex health information embedded in richer narratives the public encounters in real life. Moreover, our finding is restricted to younger adults, while middle‐aged and older adults are more susceptible to health information.

## Funding

This work was supported by Hebei Social Science Foundation Project, HB25XX005.

## Ethics Statement

The research was approved by the Ethics Committee of Qinhuangdao First Hospital. The ethical review was conducted through cooperative institutional channels, and all experimental procedures strictly followed approved ethical standards. Each participant provided the written informed consent. All methods of this study were performed in accordance with the Declaration of Helsinki.

## Conflicts of Interest

The authors declare no conflicts of interest.

## Supporting information


**Table S1:** Pairs of source and information.
**Table S2:** Items in the questionnaire of credibility and Perceived risk.
**Figure S1:** Affective reactivity. Group mean subscale scores with SE for positive affect (PA) and negative affect (NA), pre and post affect induction, respectively. ***p < 0.001, ns, non‐significant.

## Data Availability

The data that support the findings of this study are available from the corresponding author upon reasonable request.
