# Supplementary material for: Effects of Affect and Source on Adoption of Health Information
Source: Psych J. 2026 May 28;15(3):e70109. doi: 10.1002/pchj.70109 (PMC13239839; doi:10.1002/pchj.70109)
Supplement: Supplementary file 1 — Table S1: Pairs of source and information. Table S2: Items in the questionnaire of credibility and Perceived risk. Figure S1: Affective reactivity. Group mean subscale scores with SE for positive affect (PA) and negative affect (NA), pre and post affect induction, respectively. ***p < 0.001, ns, non‐significant. [file PCHJ-15-e70109-s001.docx]

**Part 1 Selection and manipulation check of materials**

*Six widely-used medical experts and six GAIs in China were selected. Twenty-five pairs of health information with opposite valence in daily life were selected from databases of authoritative medical institutions (China Public Health Network, <http://www.chealth.org.cn/).> Each pair of information was generated from the same topic. One piece of information leads to positive result, the other leads to negative result. One hundred and two participants (aged 18-25, 50 males) who did not take part in the formal experiment rated valence of information (1 = negative, 5 = positive), the familiarity of information and sources (1 = very unfamiliar, 5 = very familiar) on online 5-point Likert scales (https://www.wjx.cn). The mean valence rating of each piece of information was calculated. Twelve pairs of information were finally selected as experimental stimuli. The minimum score of positive information was higher than the maximum score of negative information. One-way ANOVA showed a significant difference in valence ratings between positive (M_positive_ = 3.827, SD_positive_ = 0.201) and negative information sets (M_negative_ = 3.043, SD_negative_ = 0.231), F(1,22)=78.674, p< 0.001. One-way ANOVA showed no significant difference in familiarity ratings between the two sets of information (F(1,22)=2.429, p = 0.133, M_positive_ = 3.173, SD_positive_ = 0.325,M_negative_= 2.991, SD_negative_ = 0.242). Three most familiar medical experts and 3 most familiar GAI were screened as the final information source stimuli. One-Way ANOVA showed no significant difference in familiarity ratings between two types of sources (F(1,4)=1.028 p = 0.368, M_medical experts_= 4.464, SD_medical experts_ = 0.062, M_GAI_ = 4.618, SD_GAI_ = 0.255). The materials used in the formal experiment are in Table 1.*

*These participants also rated the credibility of information and information sources on a 5-point Likert scale (1 = strongly disagree, 5 = strongly agree). Information source credibility scale and information credibility scale (Filieri & McLeay, 2014) are in Table 2. One-way ANOVA showed no significant difference in credibility rating of sources between two types of sources, F(1,4)=0.444, p =0.542, M_medical experts_= 4.546, SD_medical experts_ = 0.051, M_GAI_ = 4.512, SD_GAI_ = 0.071. And there was no significant difference in credibility rating between two types of information, F(1,22)=2.51, p = 0.127, M_positive_ = 4.00, SD_positive_ = 0.134,M_negative_= 4.074, SD_negative_ = 0.102.*

*To control the influence of different health topic on perceived risk, one hundred and two participants (aged 18-25, 53 males) who did not take part in the formal experiment rated perceived risk of information on an online 9-point Likert scale on the Sojump Platform(https://www.wjx.cn). Information perceived risk scale adopted from Liu et al. (2024) is in Table 2. According to the health topic classification issued by the World Health Organization (WHO) and relevant empirical research (Zhang, 2021), the health information in this study was categorized into two major topics: dietary safety and preventive health care. A two-way ANOVA was performed on perceived risk with 2 (information affect: positive/negative)×2 (health topics: food safety/preventive healthcare). The results showed that the main effect of health topics was not significant, F(1,22)=0.00, p=0.991,η_p_*² = 0.00, *M_dietary safety_=5.193, SE_dietary safety_=0.190 vs. M_preventive health care_=5.196, SE_preventive health care_=0.190. Similarly, the main effect of health information valence was also non-significant,, F(1,22)=0.295, p=0.593, η_p_*² = 0.015, *M_positive_=5.268, SE_positive_=0.190 vs. M_negative_=5.122, SE_negative_=0.190.*

Table 1. Pairs of source and information

| Source | Information  (In Chinese) | Information  (In English) | health topic of information |
| --- | --- | --- | --- |
| 主治医师Physician | 吃木耳有助于降低人体的胆固醇含量 | Black fungus helps reduce human cholesterol levels. | 预防保健preventive health care |
|  | 木耳一定程度上影响人体的凝血功能 | Black fungus affects human blood coagulation to a certain extent |  |
|  | 食用蜂蜜有助于提高人体的消化功能 | Honey consumption helps improve human digestive function. | 预防保健preventive health care |
|  | 吃蜂蜜在一定程度上会影响血糖水平 | Honey intake affects blood glucose levels to a certain extent |  |
| 主任医师Botanic Physician | 番茄红素一定程度上导致胃肠道问题 | Lycopene causes gastrointestinal problems to a certain extent | 预防保健preventive health care |
|  | 番茄红素有助于延缓人体的细胞老化 | Lycopene helps delay human cell aging |  |
|  | 喝牛奶有助于补充身体所需要的钙质 | Milk consumption helps supplement calcium required by the human body. | 饮食安全  food safety |
|  | 饮用牛奶一定程度上会引发消化不良 | Milk drinking may induce indigestion to a certain extent. |  |
| 营养专家Dietitian | 食用大蒜一定程度上导致胃肠道不适 | Garlic consumption may cause gastrointestinal discomfort to a certain extent. | 预防保健preventive health care |
|  | 大蒜有抗菌和降低人体胆固醇的作用 | Garlic has antibacterial effects and helps reduce human cholesterol |  |
|  | 饮酒在一定程度上增加心血管病风险 | Alcohol consumption increases the risk of cardiovascular diseases to a certain extent. | 饮食安全  food safety |
|  | 饮酒能促进人体血液循环和放松 | Alcohol consumption can boost blood circulation and relieve negative emotions. |  |

Table 1 (continued)

| Deepseek | 枸杞一定程度上会导致人体消化不良 | Goji berries may cause indigestion to a certain extent | 预防保健preventive health care |
| --- | --- | --- | --- |
|  | 食用枸杞有助于增强机体的免疫能力 | Goji berry consumption helps enhance human immune function. |  |
|  | 吃坚果有助于补充人体所需的蛋白质 | Nut consumption helps supplement essential proteins for the human body. | 饮食安全  food safety |
|  | 坚果一定程度上升高人体胆固醇含量 | Nut intake may elevate human cholesterol levels to a certain extent. |  |
| 文心一言Ernie Bot | 吃鳗鱼有助于补充人体蛋白质和 | Eel consumption helps supplement human protein and DHA. | 饮食安全  food safety |
|  | 鳗鱼中的脂肪含量较高会影响体重健康 | The high fat content of eels may interfere with body weight management. |  |
|  | 食用姜根一定程度上导致胃灼热问题 | Ginger root consumption may cause heartburn to a certain extent. | 饮食安全  food safety |
|  | 食用姜根有助于改善人体的血液循环 | Ginger root consumption helps improve human blood circulation. |  |
| 豆包Doubao AI | 椰子油有助于控制人体总热量的摄入 | Coconut oil helps control total daily calorie intake. | 饮食安全  food safety |
|  | 椰子油一定程度上增加血管疾病风险 | Coconut oil may increase the risk of vascular diseases to a certain extent. |  |
|  | 饮用绿茶一定程度上会影响睡眠质量 | Green tea consumption may impair sleep quality to a certain extent. | 预防保健preventive health care |
|  | 饮用绿茶有助于促进人身体的抗氧化 | Green tea consumption promotes the body’s antioxidant capacity. |  |

Table 2. Items in the questionnaire of credibility and Perceived risk

| Variable | Item(In Chinese) | Item(In English) |
| --- | --- | --- |
| Information source credibility | 我认为XXX发布的健康信息是专业的 | I regard the health information issued by XXX as professional. |
|  | 我认为XXX发布的健康信息是值得信赖的 | I regard the health information issued by XXX as trustworthy. |
|  | 我认为XXX具有较高的知识水平 | I regard XXX as having a high level of knowledge. |
| Information credibility | 我认为以下信息内容是完整的、丰富的 | I regard the following information as complete and comprehensive. |
|  | 我认为以下信息是真实的、准确的 | I regard the following information as truthful and accurate. |

Table 2 (continued)

|  | 我认为以下信息是清晰的、容易理解的 | I regard the following information as clear and easy to understand. |
| --- | --- | --- |
| Perceived risk | 请评价这条信息带来健康上严重问题的程度（1=完全不严重，9=极其严重） | Please rate the severity of health problems implied by this information. (1 = Not severe at all, 9 = Extremely severe) |
|  | 请评价这条信息危及健康的程度（1=完全不危及，9=极其危及） | Please rate the degree to which this information endangers health. (1 = Not endangering at all, 9 = Extremely endangering) |
|  | 请评价这条信息造成严重后果的程度（1=完全不严重，9=极其严重） | Please rate the seriousness of the consequences suggested by this information. (1 = Not serious at all, 9 = Extremely serious) |

**Part 2 Affect Induction**

*All videos were selected from previous research. The video used to induce positive state was selected from Wonderful Moments of Olympic Championship Victories(Xie & Yang, 2016). The video for negative state was taken from Sichuan Wenchuan Earthquake Clips. The video for neutral state was chosen from Network News Broadcast (Wang & Li, 2012) The positive and negative videos both last for 128 s. The duration of neural video is 126 s. The resolution of the videos is 1080P. The frame rate of the videos is 24 FPS.*

*Paired-samples t-tests were conducted on the subscale scores of positive affect (PA) and negative affect (NA) before and after affect induction. The positive group reported higher overall PA (t(54) =-5.516, p < 0.001, d= 0.527) and lower overall NA (t(54)=6.343, p < 0.001, d= -0.668) after induction. The negative group reported lower overall PA (t(54)=13.523, p < 0.001, d= -1.699) and higher overall NA (t(54) =-5.896, p < 0.001, d= 0.704) after induction. The neutral group showed no significant changes for either PA (t(54)=1.700, p=0.095, d= -0.191) or NA (t(54)=1.731, p=0.089, d= -0.249) after induction.These results indicate that the affect induction was effective (see Figure 1).*

*
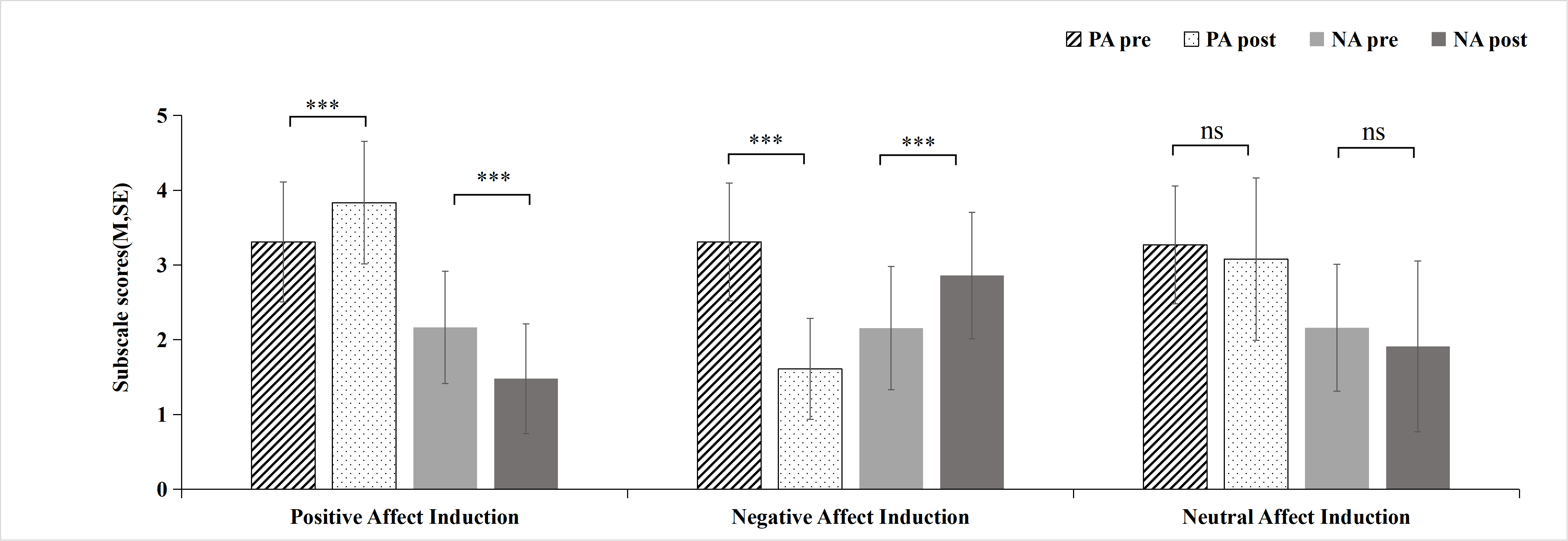
*

Figure 1. Affective reactivity. Group mean subscale scores with SE for positive affect (PA) and negative affect (NA), pre and post affect induction, respectively. ****p < .001,ns.non-significant.*

*References:*

*Filieri, R., & McLeay, F. (2014). E-WOM and accommodation: An analysis of the factors that influence travelers’ adoption of information from online reviews. Journal of travel research, 53(1), 44-57.*

*Liu, Y., Wang, X., Wang, N., & WUJl, S. (2024). A Study of Algorithmic Resistance Behavior in Social Media lnformation Dissemination under Major Public Health Emergencies. Library and Information Service, 68(09), 98-109.*

*Wang, Z., & Li, N. (2012). The Mechanism of Autonomic Nervous Activity that Underlies Positive Emotions Speeding up Recovery from Cardiovascular Reactivity. Journal of Psychological Science, 35(05), 1047-1053.*

*Xie, Y., & Yang, Z. (2016). A Comparative Study on the Validity of Different Mood Induction Procedures (MIPs). Studies of Psychology and Behavior, 14(05), 591-599.*

*Zhang, S. (2021). Study on Feature Identification of False Health Information on Social Media. Library and Information Service, 65(09), 70-78.*
